# Caffeine exposure induces browning features in adipose tissue in vitro and in vivo

**DOI:** 10.1038/s41598-019-45540-1

**Published:** 2019-06-24

**Authors:** Ksenija Velickovic, Declan Wayne, Hilda Anaid Lugo Leija, Ian Bloor, David E. Morris, James Law, Helen Budge, Harold Sacks, Michael E. Symonds, Virginie Sottile

**Affiliations:** 1 Wolfson Centre for Stem Cells, Tissue Engineering and Modelling (STEM), Division of Cancer & Stem Cells, University of Nottingham, Nottingham, NG7 2UH United Kingdom; 2 The Early Life Research Unit, Division of Child Health, Obstetrics and Gynaecology, University of Nottingham, Nottingham, NG7 2UH United Kingdom; 3 Nottingham Digestive Disease Centre and Biomedical Research Centre School of Medicine, University of Nottingham, Nottingham, NG7 2UH United Kingdom; 4 Department of Electrical and Electronic Engineering, Faculty of Engineering, University of Nottingham, Nottingham, NG7 2RD United Kingdom; 5 VA Endocrinology and Diabetes Division, VA Greater Los Angeles Healthcare System, and Department of Medicine, David Geffen School of Medicine; University of California, Los Angeles, CA 90073 USA

**Keywords:** Stem-cell research, Molecular imaging

## Abstract

Brown adipose tissue (BAT) is able to rapidly generate heat and metabolise macronutrients, such as glucose and lipids, through activation of mitochondrial uncoupling protein 1 (UCP1). Diet can modulate UCP1 function but the capacity of individual nutrients to promote the abundance and activity of UCP1 is not well established. Caffeine consumption has been associated with loss of body weight and increased energy expenditure, but whether it can activate UCP1 is unknown. This study examined the effect of caffeine on BAT thermogenesis *in vitro* and *in vivo*. Stem cell-derived adipocytes exposed to caffeine (1 mM) showed increased UCP1 protein abundance and cell metabolism with enhanced oxygen consumption and proton leak. These functional responses were associated with browning-like structural changes in mitochondrial and lipid droplet content. Caffeine also increased peroxisome proliferator-activated receptor gamma coactivator 1-alpha expression and mitochondrial biogenesis, together with a number of BAT selective and beige gene markers. *In vivo*, drinking coffee (but not water) stimulated the temperature of the supraclavicular region, which co-locates to the main region of BAT in adult humans, and is indicative of thermogenesis. Taken together, these results demonstrate that caffeine can promote BAT function at thermoneutrality and may have the potential to be used therapeutically in adult humans.

## Introduction

Brown adipose tissue (BAT) is rapidly activated by diet and cold exposure and has the potential to improve metabolic homeostasis in adults^1–3^. This adaptation is characterised by enhanced function of the BAT specific uncoupling protein 1 (UCP1), located on the inner mitochondrial membrane^4^. In adult humans, as the amount of BAT decreases with age and is negatively correlated with body mass index (BMI)^5–8^, changing the rate of BAT loss could have marked benefits for metabolic health. This could be achieved through dietary ingredients but, although it has been shown that diet can stimulate BAT function^9^, the extent to which individual nutrients can have comparable effects is not well established. Animal studies indicate that brown and beige cells could be activated through nutrients such as capsaicin analogs^10,11^, and capsinoids exert similar effects in increasing BAT-dependent energy expenditure as cold exposure^12^. Caffeine (1,3,7-trimethylxantine), a plant alkaloid found in coffee, tea, cola, and chocolate, is widely consumed, has been associated with weight loss and increased energy expenditure in both human and animal models *in vivo*, and reduces the risk of type 2 diabetes^13–18^. Although caffeine has been reported to upregulate UCP1 in obese mice^19^, the extent to which caffeine (or coffee) may directly stimulate BAT is not known. To elucidate this, we utilised a recently established stem cell model of browning^20^ to determine whether a physiological amount of caffeine could change mitochondrial function and lipid handling through promotion of UCP1 function. Based on the recent literature^21–23^, a caffeine concentration of 1 mM was used for *in vitro* cell culture. A human *in vivo* study was then undertaken to determine whether the amount of caffeine normally present in a standard coffee beverage can elicit a thermogenic effect on supraclavicular BAT in adult humans. This was assessed by an increase in temperature measured using thermal imaging of the supraclavicular region. Increases in temperature in the region is well correlated with the enhanced radiolabelled glucose uptake into BAT measured during cold exposure using positron emission tomography-computed tomography (PET-CT)^24^.

## Methods

### Cell culture

Mouse mesenchymal stem cells (mMSCs) were cultured as previously described^20^ and adipogenesis induced using a growth medium supplemented with 1 μM dexamethasone (Cayman Chemicals, USA), 100 µM isobutylmethylxanthine (IBMX) (Sigma-Aldrich, UK), 1 μM rosiglitazone (Cayman Chemicals, USA), 10 μg/ml insulin (Sigma-Aldrich, UK) and 1 nM triiodothyronine (T3) (Sigma-Aldrich, UK)). To establish the optimal amount of caffeine, mMSCs were differentiated in increasing concentrations ranging from 0.1–10 mM over 7 days. This demonstrated that the optimum concentration for viability and differentiation was 1 mM (Fig. S1) and this was, therefore, used in all subsequent experiments. A similar treatment (with an increased IBMX concentration to 500 μM) was applied to primary human bone marrow-derived stem cells (hMSCs) (Lonza, UK) over 21 days. For both cell types, cell viability assay and Oil Red O (ORO) staining were performed as previously described^20^. The analysis performed in this study was carried out on 3 batches of cells (each one done with 3 technical replicates).

### Mitochondrial staining and Immunocytochemistry

Cells cultured on coverslips were stained with 100 nM MitoTracker Deep Red FM and then examined using a Zeiss Elyra PS.1 microscope to determine the intensity of mitochondrial staining^20^. Cells were fixed and the abundance of UCP1 and proliferator-activated receptor gamma coactivator (PGC-1α) was determined using validated primary antibodies (ab10983; ab54481, Abcam, UK)^20,25–29^. Twenty fields of view from three experiments for each condition (three biological replicates) were randomly selected, and the overall fluorescence intensity for UCP1 and MitoTracker signals was measured applying identical instrument settings (scanning laser power 633 nm at 4%, detector gain set at 750 and detector offset was set to zero). Image processing and fluorescence measurements were done using original raw files on the manufacturer’s software ZEN blue 2.1 (https://www.zeiss.com/microscopy/int/products/microscope-software/zen-lite.html).

### Transmission electron microscopy analysis

Cells were fixed in pre-warm (37 °C) fixative solution (2.5% glutaraldehyde in 0.1 M cacodylate buffer, pH 7.2) and post-fixed in 1% osmium tetroxide in the same buffer following washing. Each sample was then infiltrated with a mixture of epoxy resin and propylene oxide, polymerized with resin and sectioned on a Leica UC6 ultramicrotome (Leica Microsystems, UK). For ultrastructural examination, ultrathin sections were mounted on grids, contrasted with uranyl acetate followed by lead acetate and examined using a FEI Tecnai 12 Biotwin transmission electron microscope (TEM).

### Gene expression analysis

Extraction of total RNA and quantitative PCR were performed as previously described^20^. Gene expression was determined using the GeNorm normalization algorithm against two selected reference genes (stability value M = 0.28), beta-2-microglobulin (B2M) and acidic ribosomal protein subunit P0 (RPLP0) using GeNorm software (version 3.5; Primer Design Ltd). UCP1, COX8b, P2RX5, DIO2, ARß3, ARα2, TRPV1, TRPV2 and TRPV4 gene expression was determined using TaqMan probe (BioRad TaqMan Gene Expression assays qHsaCEP0050537, qMmuCIP0034367, qRnoCIP0024301, qMmuCEP0052679 qMmuCEP0031789, qMmuCEP0055983, qMmuCIP0031313, qMmuCIP0035343 and qMmuCIP0032629, respectively). Murine-specific oligonucleotide primers (Eurofins) are given in Supplementary Table 1. A principal component analysis (PCA) and heat map were constructed to evaluate the overall changes of caffeine on adipocyte differentiation using ClustVis^30^. For qPCR analysis, 5 different passages treated and analysed independently were used.

### Seahorse assay

Cells were seeded into the XFe96 Microplates (Seahorse Bioscience) to measure the oxygen consumption rate (OCR: an indicator of mitochondrial respiration) and the extracellular acidification rate (ECAR: an indicator of glycolysis) as previously described^20^. OCR and ECAR readings were measured under basal conditions and after the addition of mitochondrial inhibitors (oligomycin, carbonyl cyanide-4-(trifluoromethoxy) phenylhydrazone (FCCP) and rotenone/antimycin A). Coupling efficiency, an indicator of the proportion of respiratory activity used to make ATP, was determined by calculating the percentage of OCR immediately following the oligomycin treatment with the final baseline value. Seahorse datasets were normalised to cell number using Hoechst 33258 fluorescent dye signal measured immediately after Seahorse analysis^31^.

### Human *in vivo* response to caffeine

Healthy volunteers (4 male; 5 female), aged 27 (SD 6) years of normal BMI (mean 23 (SD 3) kg/m^2^), were imaged in standard loose fitting clothing to reveal the region of interest, in the morning, without prior vigorous exercise, caffeine, drug or alcohol consumption within 9 hours and at least 2 hours after eating. Thermally-reflective skin markers were placed on precise anatomical landmarks for subsequent data analysis^32^. Imaging was undertaken within a temperature controlled room (maintained at 22 °C; external ambient temperatures on study days: 16.8 (SD 2.4) °C). Participants acclimatised for 30 minutes prior to imaging before baseline imaging (FLIR T540, FLIR Systems AB, Danderyd, Sweden)) as previously described^32^. After baseline imaging, participants consumed either a caffeinated beverage (Nescafe^©^ Original 1.8 g sachet ~65 mg caffeine dissolved in 200 ml water at 22 °C; n = 9) or water alone at 22 °C (n = 9), remained seated for 30 minutes (to allow time for caffeine absorption^33^) and then underwent further thermal imaging. The study was conducted according to the standards of Declaration of Helsinki following approval of the University of Nottingham Medical School Ethics Committee and with informed participant consent. Nine participants in each group gave our study 95% power to detect a biologically significant mean difference of 0.2 °C^24^.

Thermal images were analysed as described previously with the hottest 10% of the points within the thermal image identified and the medians of those points calculated^32^. Mean pre-stimulation supraclavicular temperature (T_SCV_) was calculated 20 minutes prior to ingestion of coffee (n = 9) or water (n = 9), whilst mean post-stimulation T_SCV_ was measured from 30 minutes after ingestion.

### Statistical analyses

All data were analysed by Student’s t test. For data that was not normally distributed the nonparametric Mann Whitney test was used to evaluate the statistical significance between two treatment groups. Statistical significance was accepted at P < 0.05, with *P < 0.05; **P < 0.01; ***P < 0.001. Errors bars plotted on graphs are presented as the mean ± SEM. Data were analysed using Graph Pad Prism Software (https://www.graphpad.com). For principal component analysis, Metaboanalyst (https://www.metaboanalyst.ca) was used.

## Results

### Caffeine treatment increased UCP1 protein expression, mitochondrial biogenesis and bioenergetics profile in MSCs-derived adipocytes

**Figure 1:**
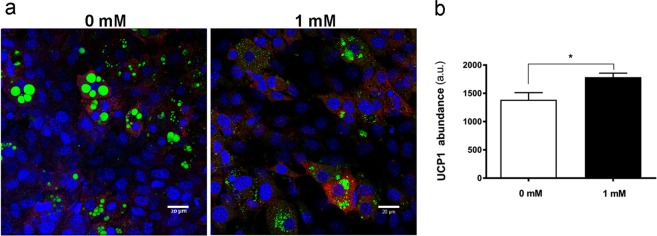
Effect of caffeine on immunofluorescence analysis of UCP1 abundance in adipogenic cultures. (**a**) Representative images showing upregulation of UCP1 (red) in mMSCs. DAPI was used to identify cell nuclei (blue) and BODIPY was used to identify lipid droplets (green). (**b**) Mean relative UCP1 abundance (n = 20). Scale bars: 20 μm; *P < 0.05.

**Figure 2:**
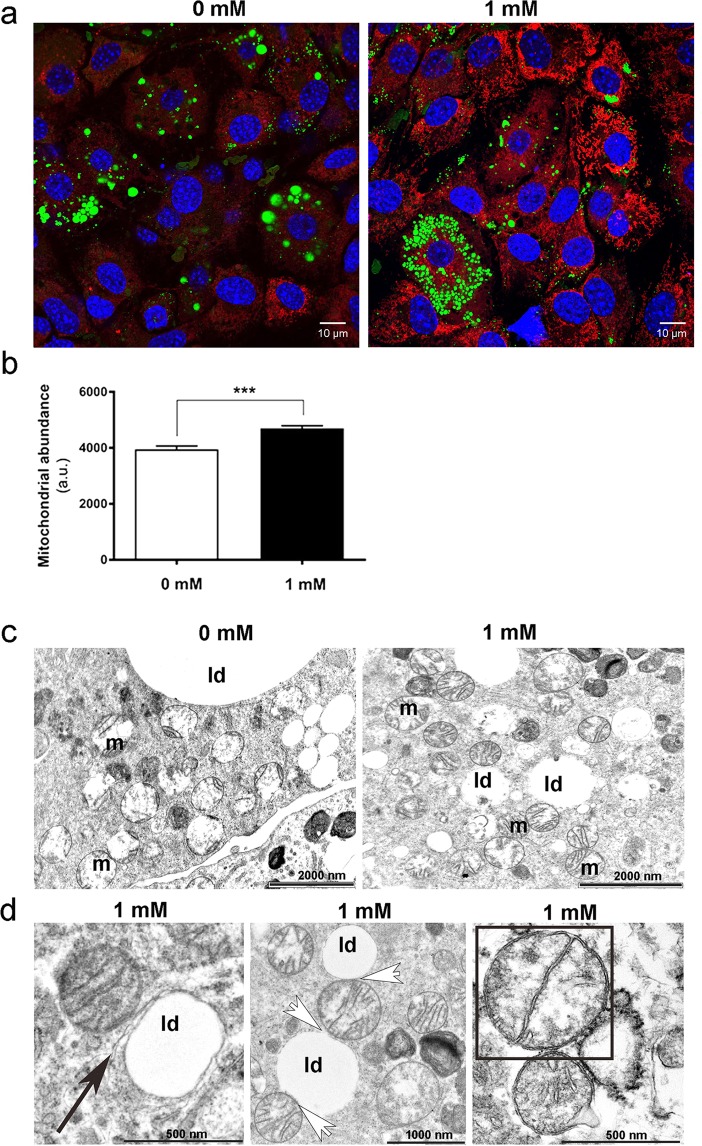
Adaptations in the mitochondrial compartment of adipocytes following 1 mM caffeine treatment. (**a**) Representative images showing mitochondrial staining (MitoTracker, red). DAPI was used to identify nuclei (blue) and BODIPY was used to identify lipid droplet (green). (**b**) Relative MitoTracker fluorescence intensity. (**c**,**d**) TEM analysis of mitochondria (m) and lipid droplets (ld), with (**d**) showing higher magnification views of caffeine-treated cells with contact sites between lipid droplets and endoplasmic reticulum (black arrow), contact sites between mitochondria and lipid droplets (white arrows), and mitochondria division (outlined square). Scale bar: 10 μm. ***P < 0.001.

**Figure 3:**
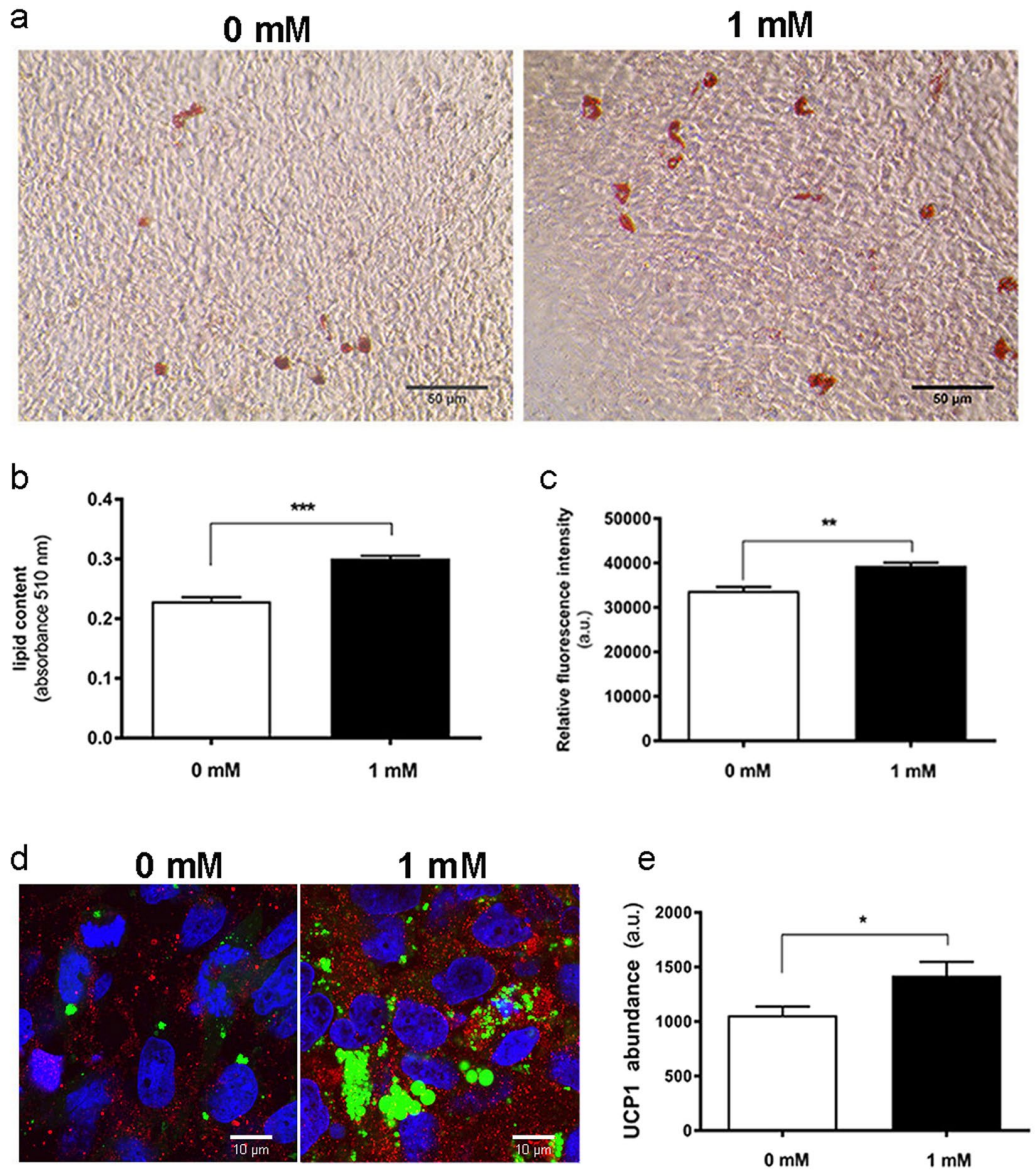
hMSC response to caffeine exposure during a 21-day period of adipogenic differentiation. (**a**) Representative images of cells stained for lipid droplets with ORO (Scale bar: 50 μm) and mean (**b**) staining quantified spectrophotometrically. (**c**) Cell viability assay. (**d**) Representative images showing upregulation of UCP1 (red) in hMSCs. DAPI was used to identify cell nuclei (blue) and BODIPY was used to identify lipid droplets (green); scale bar: 10 μm and (**e**) mean relative UCP1 abundance (n = 20). Data presented as mean ± SEM; *P < 0.05; **P < 0.01; ***P < 0.001.

Caffeine promoted the expression of UCP1 in mMSC cultures (Fig. 1a,b), a response accompanied by enhanced abundance of peroxisome proliferator-activated receptor gamma coactivator (PGC)-1α, with the nuclear presence of PGC-1α seen in most adipocytes exposed to caffeine, contrary to controls (Fig. S2). Mitochondrial staining further indicated that caffeine exposure enhanced mitochondriogenesis (Fig. 2a,b). Ultrastructural TEM analysis revealed more numerous and densely packed mitochondria with caffeine treatment, in an interconnected reticular pattern (Fig. 2c). The number of mitochondria undergoing division was enhanced by caffeine which also caused modification in their shape, resulting in a more rounded appearance with abundant lamellar cristae. These adaptations were accompanied with enhanced contact sites between mitochondria and lipid droplets, and between lipid droplets and endoplasmic reticulum (Fig. 2d). Comparable responses were observed in human MSC cultures, with caffeine causing an increase in lipid content, metabolic activity and UCP1 protein abundance (Fig. 3).

**Figure 4:**
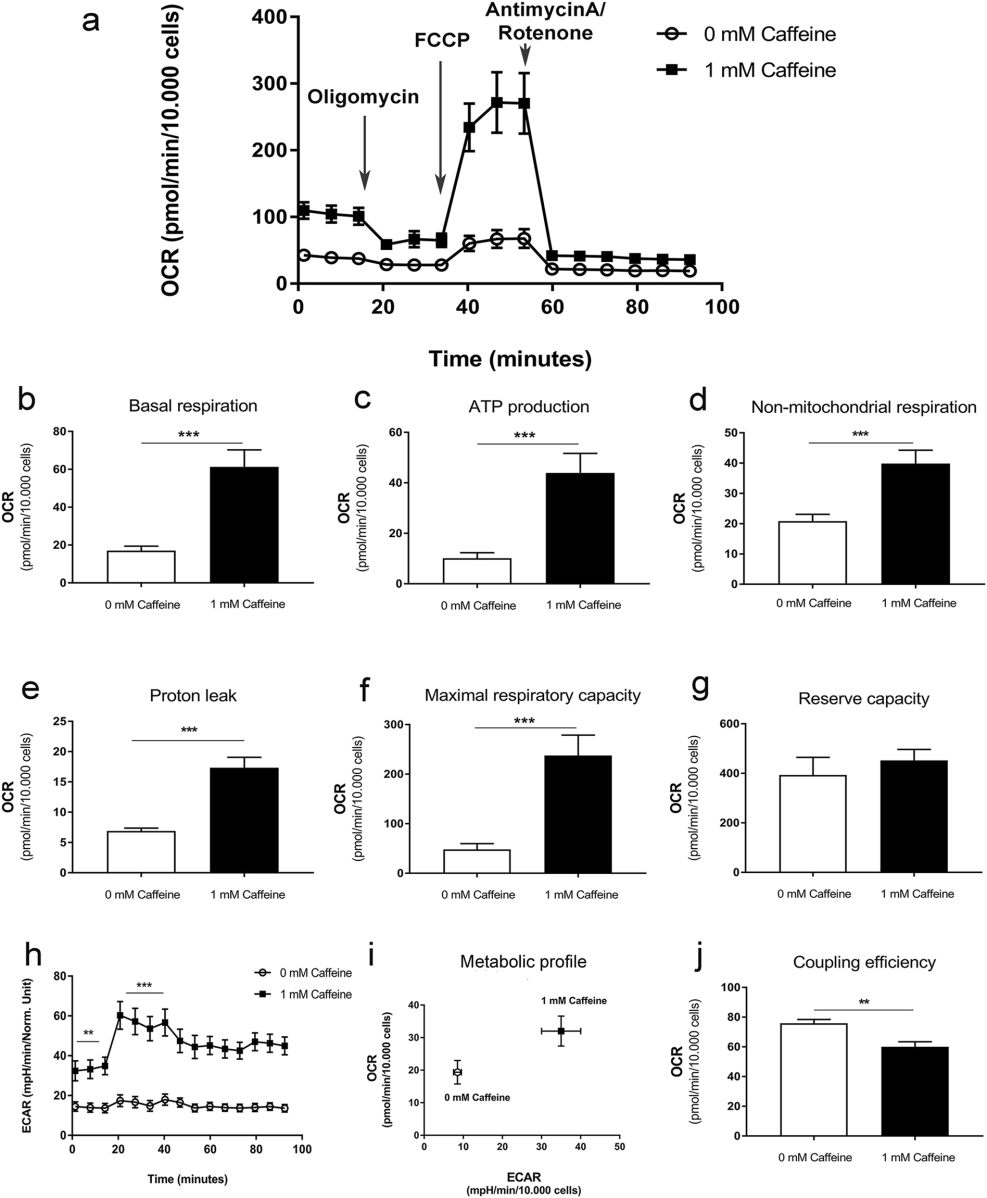
Seahorse XF cell mitochondrial stress test assay performed on mouse mesenchymal stem cell-derived adipocytes treated with or without caffeine for 7 days. (**a**) OCR profile plot. (**b**) Basal respiration. (**c)** ATP production. (**d**) Non-mitochondrial respiration. (**e**) Proton leak. (**f**) Maximal respiratory capacity. (**g**) Reserve capacity. (**h**) ECAR. (**i**) Summary metabolic profiles as determined by plotting ECAR against OCR. (**j**) Coupling efficiency. Histograms represent mean ± SEM. n = 3; **P < 0.01, ***P < 0.001.

To determine whether the observed caffeine-induced changes in mitochondrial biogenesis were accompanied by functional changes in bioenergetics, cultures were analysed using the Seahorse assay. This demonstrated that caffeine enhanced basal respiration, respiration used for the ATP-production, non-mitochondrial respiration, proton leak and maximal respiratory capacity (Fig. 4a–f). Basal ECAR was also raised by caffeine both before, and after, oligomycin treatment (Fig. 4h), and when plotted against OCR (Fig. 4i). Caffeine increased glycolysis and oxidative phosphorylation, indicative of more metabolically active cells, as well as decreasing coupling efficiency (Fig. 4j).

### Caffeine treatment shows upregulation of beige/brown genes during MSC adipogenesis

**Figure 5:**
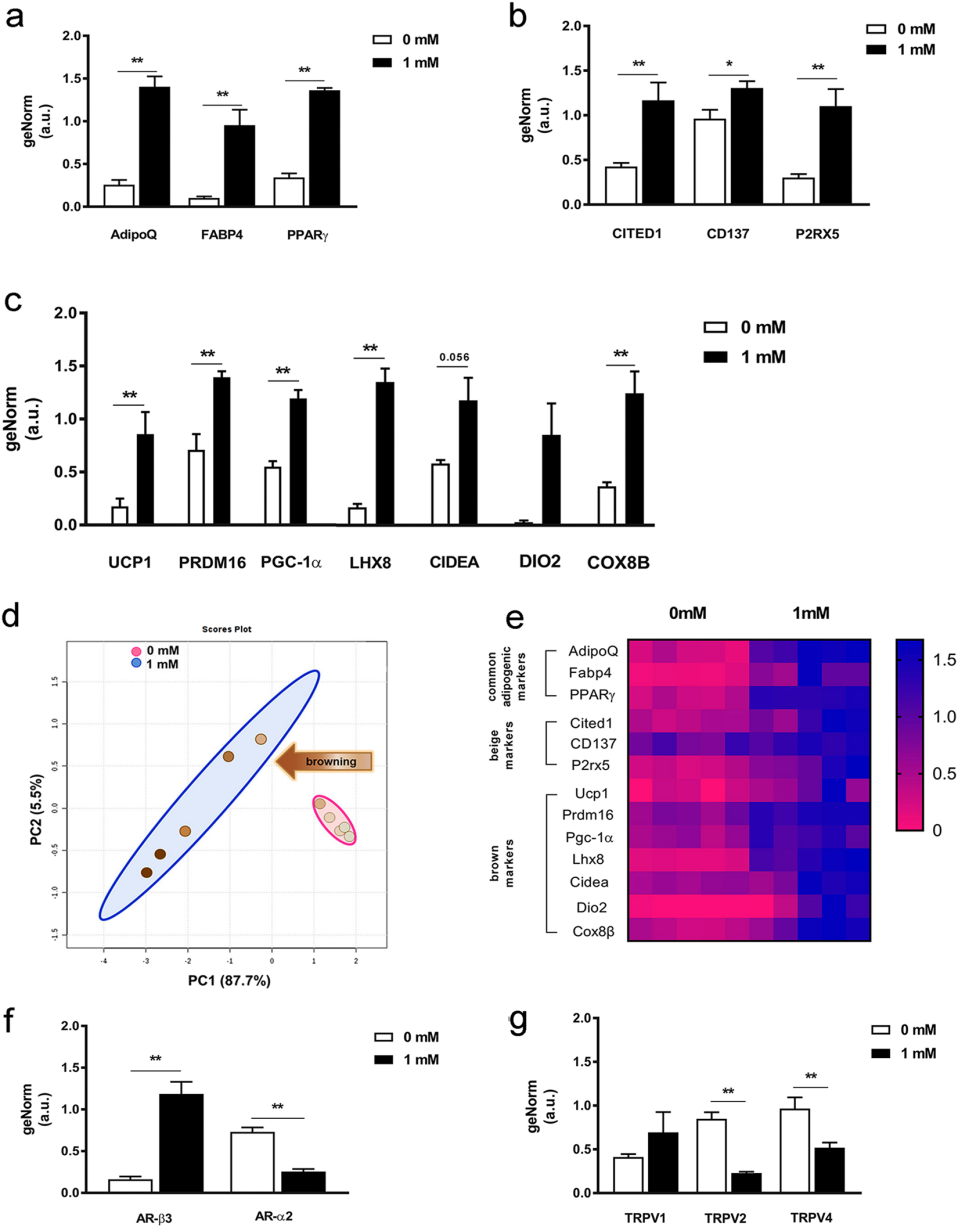
Adipogenic gene expression analysis by qPCR during mMSCs differentiation treated with or without caffeine. Relative gene expression of (**a**) adipogenic markers AdipoQ, FABP4 and PPARγ, (**b**) beige lineage markers CITED1, CD137 and P2RX5, and (**c**) brown lineage markers UCP1, PRDM16, PGC-1α, LHX8, CIDEA, DIO2 and COX8b. (**d**) Scatter plot of the first two principal components, comprising 93.2% of total variance, highlighting two clusters corresponding to cells differentiated in adipogenic medium with 0 mM (pink) and 1 mM (blue) caffeine. (**e**) Unsupervised hierarchical clustering of expression values for the same genes and samples as those in panel D. Each row represents a gene while each column corresponds to a different sample. Gene expression of (**f**) AR-ß3 and AR-α2 and (**g**) TRPV1, TRPV2 and TRPV4. Data represent the mean ± SEM of five replicates; *P < 0.05; ***P < 0.001.

Caffeine increased gene expression of PPARγ, adiponectin and FABP4 (Fig. 5a), as well as beige markers CITED1, CD137 and P2RX5 (Fig. 5b) and brown-selective genes UCP1, PRDM16, PGC-1α, LHX8 and COX8b (Fig. 5c) and AR-ß3 (Fig. 5f). PCA and heat maps further revealed separate gene clusters, visually confirming discrete expression patterns with caffeine (Fig. 5d,e), which also decreased the abundance of AR-α2 (Fig. 5f) TRPV2 and TRPV4 mRNA (Fig. 5g). Raw Ct-values for UCP1 gene expression are presented in Supplementary Table 2.

### Drinking coffee stimulates the temperature of the supraclavicular region co-locating with BAT in adults

**Figure 6:**
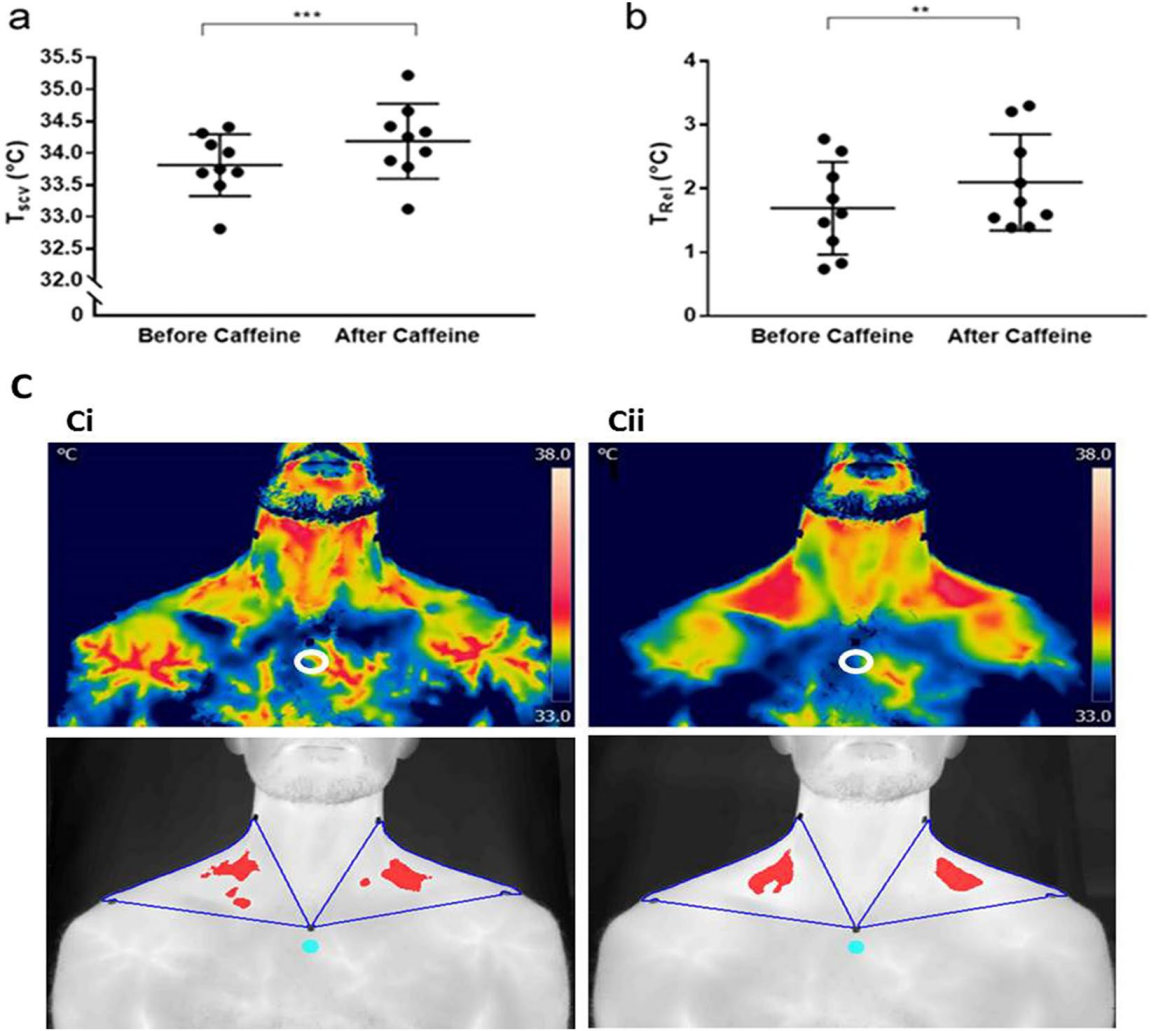
*In vivo* effect of drinking caffeine on heat production from brown fat in adult humans. (**a**) Caffeine resulted in a significant rise in temperature of the supraclavicular region (T_SCV_) which co-locates with brown fat and this reflects (**b**) increase in supraclavicular temperature relative to reference body surface temperature (T_rel_) (n = 9); (**c**) Representative thermal image (i) pre and (ii) post-caffeine, as either the original FLIR image, or the transformed image high-lighting the hottest 10% of pixels. ***P < 0.001; **P < 0.01. White open and blue closed circles indicate the reference temperature point.

Drinking coffee significantly increased the temperature of the supraclavicular region co-locating with brown adipose tissue in humans^23^ (Fig. 6), reflecting increasing T_SCV_ relative to reference body surface temperature (T_rel_;), an effect not seen with consuming water alone (T_SCV_: before water ingestion: 34.55 ± 0.16 °C; after water ingestion: 34.55 ± 0.17 °C; ΔT_REL_ with water ingestion: 0.13 ± 0.12 °C; n = 9). The temperature of the reference point was unchanged e.g. pre caffeine 31.12 ± 0.27 °C; post caffeine 32.09 ± 0.31 °C (n = 9).

## Discussion

This is the first study to determine that the stimulatory effects of caffeine on UCP1 seen *in vitro* can be translated to adult humans ingesting caffeine in a commonly consumed coffee beverage. The increase in temperature of the region which co-locates with BAT observed with caffeine ingestion is indicative of an increase in BAT activity^32^ following a relatively low dose of caffeine^33^ from a single standard cup of coffee. It is supportive of our *in vitro* observations in human and murine stem-cell derived adipocytes, in which the abundance of UCP1 was increased, together with oxygen consumption, and markers of mitochondrial and lipid metabolism. These findings suggest that the increase in metabolic rate following caffeine ingestion could be mediated by enhanced BAT function, which precedes any change in skin temperature^34^.

Enhanced BAT activity in adult humans has the potential to improve lipid and carbohydrate homeostasis as well as contributing to weight loss strategies^1^. The extent to which individual components of the diet can activate BAT in humans is not well established, as the gold standard to assess BAT function is the uptake of radio-labelled glucose (fludeoxyglucose or ^18^FDG) as measured by PET-CT^5,8^. Normally, this has to be undertaken in fasted subjects, as feeding is accompanied by such a large increase in glucose uptake by muscle that the capacity to detect BAT is greatly reduced^35^. We have, therefore, developed thermal imaging as a practical method to detect *in vivo* changes in BAT activity and which can detect changes in response to ingestion without requiring exposure to radiation and which correlates well with the uptake of ^18^FDG following cold exposure^24^. Moreover, there is increasing evidence that dietary ingredients promote brown fat function^9^, responses which may be mediated by the release of secretin from the gut^27^.

It has been suggested that thermal imaging may not give an optimal measure of BAT^36,37^, however, such studies have not considered the changes in temperature of the supraclavicular region with an appropriate reference point that correlate well with BAT function as assessed by PET-CT^24^. In addition, single point measures of supraclavicular temperature can be misleading as they cannot reliably detect the hottest region of brown fat as identified with thermal imaging approach used here^38^. Although caffeine has been reported to cause changes in blood flow^39–41^, this was not expected to cause substantial changes in skin temperature, as this typically does not occur until at least 2 h after caffeine consumption^34^. Further studies measuring the kinetics of caffeine-induced blood flow changes can help refine its impact on thermal analysis.

Temperature, together with intact innervation^42^ are primary factors regulating the magnitude of change in brown and/or beige fat function, which is further modulated by the relative amount of UCP1 within a depot. Consequently, transferring mice from 22 to 5 °C results in a modest rise within interscapular BAT, but a much larger increase within inguinal beige fat^43^. It is thus likely that comparatively small changes in the amount of UCP1 can impact on energy balance, a proposal supported by the present study. We demonstrate that in beige adipocytes^20^, in which caffeine exposure increased the amount of UCP1 by ~20%, in conjunction with more mitochondria and lipid, this was accompanied by a marked rise in oxygen consumption *in vitro*. The magnitude of change was, however, lower than that seen when comparing mitochondria sampled from perirenal fat in humans with pheochromocytoma patients living in a warm climate^44^. It would thus be useful to further examine the extent to which comparable outcomes are found *in vitro* in brown as opposed to beige adipocytes.

Previous studies reported that caffeine inhibits differentiation, promotes lipolysis and suppresses intracellular lipid accumulation in adipocyte cultures^21,45–47^, while increasing BAT temperature, guanosine-5′-diphosphate (GDP) binding and oxygen consumption in mitochondria in mice^48^. The present study found that caffeine used at 1 mM upregulated not only UCP1 but also primary regulatory genes including PPARγ, PRDM16, and PGC-1α^49^. Caffeine, therefore, activated the main regulators of brown adipocyte thermogenesis^50–52^, but also gene expression of CD137, LHX8, P2RX5, CITED1 and COX8b, that are indicative of molecular conversion of white/beige cells into brown adipocytes. Consistent with these molecular changes induced by caffeine was the appearance of smaller lipid droplets in UCP1-expressing adipocytes, a major morphological sign of lipolysis^49^. The response to caffeine exposure was observed to vary in human and mouse cultures, in line with the different level of lipid accumulation noted under adipogenic induction in these models, pointing to intrinsic differences which will require further characterization. The recruitment of thermogenesis results in mitochondriogenesis, a process controlled by PGC-1α^50,53,54^, and was here accompanied by increased PGC-1α mRNA expression and PGC-1α protein nuclear localization indicating PGC-1α functional activity in caffeine-treated cells. In line with these observations, increased mitochondrial biogenesis and PGC-1α expression after caffeine treatment have been reported in other cell types^55–57^. PGC-1α not only triggers mitochondrial biogenesis, but also activates several components of the adaptive thermogenic program in BAT, including fatty-acid oxidation, and increases oxygen consumption through co-activation of transcription factors such as PPARγ^53^. These molecular adaptations with caffeine exposure were accompanied by changes in mitochondrial ultrastructure and the redistribution of adjacent small lipid droplets. The numerous lamellar cristae observed in caffeine-treated adipocytes are characteristic of activated beige/brown adipocytes that expand the surface of their inner mitochondria membrane with enhanced functional UCP1^4^. These contact sites constitute an important way of inter-organelle communication^58^ in which fatty acids liberated by lipolysis could be used for β-oxidation. Future analysis of sorted lipid-laden cells could provide a more specific measure of the mitochondrial response to caffeine treatment. The TRPV receptor family can also modulate beige/brown adipocyte thermogenesis, and TRPV4 can be a negative regulator of PGC1α^59^, so caffeine might further induce PGC1α and UCP1 expression by antagonizing TRPV4. The capacity of caffeine-treated adipocytes to undergo mitochondrial uncoupling was confirmed by enhanced bioenergetic capacity accompanied with an increase in ATP-producing metabolic pathways, i.e. oxidative phosphorylation and glycolysis which are indicative of higher energy demands observed during the browning process^20^, although additional pathways besides UCP1-dependent mechanisms could also be involved. The low Ct values observed here for UCP1 may be consistent with this, and might also underline differences in between the half-life of UCP1 protein (2–20 days) and UCP1 mRNA (3 h)^60,61^.

Besides acting as an adenosine receptor antagonist, caffeine inhibits phosphodiesterase, increasing intracellular cAMP^62^, a process also activated by β-adrenergic stimulation^4,63^. This could be the mechanism by which caffeine enhances UCP1 function. Coupled to G proteins, β3-AR induces cAMP formation, which in turn activates protein kinase A and stimulates fat hydrolysis, with the free fatty acids released activating UCP1^4,64^. Adenylyl cyclase (which synthesizes cAMP from ATP^65^) is positively coupled to β3-AR but inhibited by ARα2^66^, which was observed here to decrease with caffeine exposure. Taken together, the morphological evidence of lipolysis, plus reduced ARα2 gene suppression induced by caffeine, supports an antilipolytic effect^67^. Further experiments investigating the effects of PKA inhibition could provide additional information on the role of cAMP levels in the caffeine response.

In conclusion, these results provide new complementary *in vitro* and *in vivo* evidence that caffeine (and a coffee beverage) can promote BAT function at doses compatible with human use. Similar approaches could be considered to screen other potential dietary compounds that could target UCP1 and promote BAT function. Future intervention studies can now be undertaken to assess whether caffeine-induced BAT activation in humans is dose-dependent, refine the minimal intake required for a BAT response, and explore whether comparable effects are seen in fully differentiated adipocytes and primary cells, as well as in diabetic and/or obese individuals.

## Supplementary information

Suppl Info

Supplementary Dataset 2
